# Regenerative Medicine in Rotator Cuff Injuries

**DOI:** 10.1155/2014/129515

**Published:** 2014-08-13

**Authors:** Pietro Randelli, Filippo Randelli, Vincenza Ragone, Alessandra Menon, Riccardo D'Ambrosi, Davide Cucchi, Paolo Cabitza, Giuseppe Banfi

**Affiliations:** ^1^Dipartimento di Scienze Biomediche per la Salute, Università degli Studi di Milano, Via Mangiagalli 31, 20133 Milan, Italy; ^2^IRCCS Policlinico San Donato, Via Morandi 30, San Donato Milanese, 20097 Milan, Italy; ^3^IRCCS Istituto Ortopedico Galeazzi, Via Galeazzi 4, 20161 Milan, Italy

## Abstract

Rotator cuff injuries are a common source of shoulder pathology and result in an important decrease in quality of patient life. Given the frequency of these injuries, as well as the relatively poor result of surgical intervention, it is not surprising that new and innovative strategies like tissue engineering have become more appealing. Tissue-engineering strategies involve the use of cells and/or bioactive factors to promote tendon regeneration via natural processes. The ability of numerous growth factors to affect tendon healing has been extensively analyzed *in vitro* and in animal models, showing promising results. Platelet-rich plasma (PRP) is a whole blood fraction which contains several growth factors. Controlled clinical studies using different autologous PRP formulations have provided controversial results. However, favourable structural healing rates have been observed for surgical repair of small and medium rotator cuff tears. Cell-based approaches have also been suggested to enhance tendon healing. Bone marrow is a well known source of mesenchymal stem cells (MSCs). Recently, *ex vivo* human studies have isolated and cultured distinct populations of MSCs from rotator cuff tendons, long head of the biceps tendon, subacromial bursa, and glenohumeral synovia. Stem cells therapies represent a novel frontier in the management of rotator cuff disease that required further basic and clinical research.

## 1. Introduction

Rotator cuff lesions represent the vast majority of shoulder injuries in adult patients and are a common contributing factor to shoulder pain and occupational disability.

The incidence of this condition is increasing along with an aging population [1]. The management of rotator cuff tears is complex and multifactorial. Operative treatment allows primary repair to be performed either as an open or arthroscopic procedure.

Improvements in arthroscopic instrumentation and suture anchor technology have allowed the development of stronger constructs with multiple suture configurations, allowing repair of large and massive tears through minimally invasive means. However, although repair instrumentation and techniques have improved, healing rates have not. A high failure rate remains for large and massive rotator cuff tears [2, 3].

A recent meta-analysis has shown that the development and introduction of novel surgical techniques are not related to an improvement of clinical and anatomical results over the investigated period (1980–2012) [4].

To enhance tendon tissue regeneration, new biological solutions including growth factors, platelet-rich plasma (PRP), and stem cells are being investigated.

This review will outline the current evidence for the novel frontier in the management of rotator cuff disease including growth factor and stem cell therapy.

## 2. Growth Factors

Growth factors are signal molecules involved in the control of cell growth and differentiation and are active in different phases of inflammation. They are produced by inflammatory cells, platelets, and fibroblasts.

Rotator cuff healing occurs via a sequence of inflammation, repair, and remodeling [21]. Several growth factors released in the repair phase act in both an autocrine and paracrine fashion to promote cellular proliferation and matrix deposition. These include basic fibroblast growth factor (bFGF), bone morphogenetic proteins 12, 13, and 14 (BMP-12,13,14), vascular endothelial growth factor (VEGF), platelet derived growth factor (PDGF-*β*), transforming growth factor-beta (TGF-*β*), and insulin-like growth factor-1 (IGF-1) [22, 23].

Because rotator cuff healing results in reactive scar formation rather than a histologically normal insertion site, addition of these factors may enhance the repair-site biology.

PDGF is a basic protein composed of two subunits, an A and a B chain, that exists in three main isoforms (PDGF-AA, PDGF-BB, and PDGF-AB). These isoforms function as chemotactic agents for inflammatory cells and help to increase type I collagen synthesis and induce TGF-*β*1 expression [22]. The homodimer PDGF-BB has been the subject of most research because it stimulates both matrix synthesis and cell division [24, 25]. Kobayashi et al. [23] studied the expression of PDGF-BB during the early healing of the supraspinatus tendon in New Zealand white rabbits. They found that the highest concentration of PDGF-BB occurred between days 7 and 14. This period coincides with the terminal part of the inflammatory phase of tendon healing and the early part of the repair phase.

Several studies have examined the role of PDGF as a mitogenic and chemotactic cytokine that can enhance tendon and ligament healing.

Uggen et al. [26] showed restoration of normal crimp patterning and collagen-bundle alignment in a rat rotator cuff repair model after delivery of cells expressing PDGF-BB on a polyglycolic acid scaffold, compared with controls. Later, the same authors examined the effects of recombinant human PDGF-BB-coated sutures on rotator cuff healing in a sheep model [27]. This study showed enhanced histological scores of the treatment group in comparison with controls at a follow-up period of 6 weeks; however, there was no significant difference between the groups in terms of ultimate load-to-failure.

A similar study used an interpositional graft composed of a type I collagen matrix, enriched with recombinant human PDGF-BB (rhPDGF-BB), implanted in an ovine model for rotator cuff repair [28]. At 12 weeks after repair, the interpositional graft at low and medium dosages of rhPDGF-BB (75 and 150 mg) had improved biomechanical strength and anatomic appearance compared with the control group and the 500 mg rhPDGF-BB group. These studies highlight again the importance of PDGF dosing, timing, and delivery methods. Although the exact answer still remains unclear, PDGF augmentation holds promise for augmenting tendon-to-bone healing.

TGF-*β* is a family of cytokines that includes three isoforms (TGF-*β*1, 2, and 3). Although this superfamily is responsible for numerous physiologic effects, it is of particular interest in biological augmentation, as it is thought to play an important role in tendon and ligament formation through cellular proliferation, differentiation, and matrix synthesis [24].

Of the three isoforms, TGF-*β*3 holds the greatest promise to enhance the local microenvironment of the rotator cuff repair site because its high expression during fetal wound healing correlates with no formation of scar tissue [29]. By contrast, postnatal wound healing is characterized by extensive scar formation, with high levels of TGF-*β*1 and TGF-*β*2 expression and low level of TGF-*β*3. Because TGF-*β*3 is not present within the adult healing environment, its exogenous application could promote a scarless regenerative healing process. Based on this assumption, three recent studies have specifically examined the use of TGF-*β*3 in a rat rotator cuff model [29–31].

Kim et al. [30]. used an osmotic pump delivery system to specifically investigate the role of TGF-*β*1 and TGF-*β*3 at the tendon-to-bone insertion of repaired rat supraspinatus tendons. The TGF-*β*1 group showed increased type III collagen production compared with control shoulders, which is consistent with a scar-mediated healing response. There was also a trend toward reduced mechanical properties within this group. By contrast, the TGF-*β*3 group showed no histological or biomechanical differences compared with the paired controls. While these results support the TGF- *β*1 mediated reparative healing response, the lack of improvement in the TGF-*β*3 group could be attributed to the delivery system used.

A subsequent study by the same group using a heparin-/fibrin-based TGF-*β*3 delivery system to the tendon-to-bone insertion demonstrated accelerated healing with increased inflammation, cellularity, vascularity, and cell proliferation at early time points [29]. In addition, there were significant improvements in structural and mechanical properties, compared with controls. These findings suggest that TGF-*β*3 can enhance tendon-to-bone healing* in vivo* in a rat model. In a further study the authors examined the delivery to the tendon-bone interface of repaired rat supraspinatus tendons of TGF-*β*3 in an injectable calcium-phosphate matrix (Ca-P) [31]. The authors found new bone formation, increased fibrocartilage, and improved collagen organization with the use of the osteoconductive Ca-P matrix alone. With the addition of TGF-*β*3 to the Ca-P matrix, there was a significant improvement in strength at the repair site 4 weeks postoperatively and a more favorable collagen type I/III ratio, which reflects more mature healing. These results further support the role of TGF-*β*3 in improving tendon healing after surgical repair; however, future studies are needed to optimize delivery techniques and dosing as well as to examine the effects of multiple growth factors administration on the healing process.

## 3. PRP

The use of PRP as a biological solution to improve rotator cuff tendon healing has gained popularity over the last several years.

PRP is a whole blood fraction containing high platelet concentrations that, once activated, provides a release of various growth factors which participate in tissue repair processes [32].

*In vitro *studies on the effect of PRP on human tenocytes from rotator cuff with degenerative lesions showed that growth factors released by platelets may enhance cell proliferation of tenocytes and promote the synthesis of extracellular matrix [33, 34].

PRP not only inhibits the inflammatory effects of interleukin 1*β* (IL-1*β*) but also enhances TGF-*β* production. Increased concentration of IL-1*β* is significantly correlated with rotator cuff tendon degeneration; conversely, TGF-*β* enhances rotator cuff tendon repair strength [35]. Furthermore, an* in vivo* animal study showed that different types of application did not influence the effect of PRP on rotator cuff healing [36].

### 3.1. PRP Formulation

There are several different PRP formulations currently available. PRP can be classified into four main categories: pure PRP (P-PRP), leucocyte-rich PRP (L-PRP), pure platelet-rich fibrin (P-PRF), and leucocyte-rich platelet-rich fibrin (L-PRF). In each category, platelet concentration can be obtained by different processes, either in a fully automatized setup or by manual protocols [37].

Among PRP formulations, a further division can be made between those which are activated* ex vivo* with thrombin and/or calcium and those unactivated, which rely on* in vivo* activation via endogenous collagen [38].

The role of leucocytes in PRP is a controversial issue in the literature.

Basic science studies showed that growth factors and cytokine concentrations are influenced by the cellular composition of PRP, with leukocytes increasing catabolic signaling molecules [39].

Furthermore L-PRP has been found more proinflammatory when injected in rabbits [40] and increased the levels of MMPs when assayed in tenocyte cultures compared with pure PRP [41]. However, other studies have pointed out the positive role of leucocytes in PRP as anti-infectious and immune regulatory agents [42–45].

Most clinical studies have used numerous different PRP formulations. The obtained results have never been analyzed using the leucocyte content of the final concentrate as a key parameter. Thus, differences between P-PRP and L-PRP preparations are still unknown.

However the leukocyte content does not seem to induce negative effects or to impair the potentially beneficial effects of PRP and no uncontrolled immune reactions of L-PRPs have been also reported; on the contrary, the use of L-PRP could diminish pain and inflammation of the treated sites [46, 47]. Further randomized controlled trials comparing the effectiveness of L-PRP versus P-PRP will help in defining the optimal PRP formulation to manage rotator cuff injuries.

### 3.2. Surgical Use of PRP in Arthroscopic Rotator Cuff Repair

Literature showed that PRP can be applied either by direct injection or by application of a PRP matrix scaffold on repaired tissues. The main characteristics of controlled clinical studies using PRP in arthroscopic rotator cuff repair are reported in Table 1 [5–18].

Conflicting results on the effectiveness of PRP use in rotator cuff tendon repair were produced, making it now difficult to draw definitive conclusions.

The clinical studies published to date have different experimental designs with a level of evidence that varies from 1 to 4. Moreover, there are differences in PRP formulations in terms of growth factor concentration and catabolic enzyme content [39]. A PRP classification system exists, which is based on whether white blood cells are present and whether PRP is used in an activated (*ex vivo* activation with thrombin and/or calcium) or unactivated form (*in vivo *activation via endogenous collagen) [48].

Experimental protocols present differences among the trials, concerning volume of autologous blood collected, speed and time of centrifugation, method of administration, activating agent, presence of leukocytes, final volume of PRP, and final concentration of platelets and growth factors. The surgical technique (transosseous equivalent, single, or double row) and the rehabilitation protocol (standard or rapid) were not the same among different studies.

In spite of the differences in surgical techniques, PRP formulation, size of the lesions, retear rate have been recalculated by combining the available data from studies in order to determine the role of PRP in improving the rotator cuff healing after surgical repair.

Differences in term of retear rate between PRP and control group were assessed by a chi-square test. The analysis of all studies examined showed that there was no significant difference in the retear rate between PRP and control group. The retear rate was 31% (101 out of 323) and 37% (115 out of 312), respectively (*P* value > 0.05).

A significant difference was found when a stratified analysis was performed to analyze the results of small and medium lesions of the rotator cuff. The rate of reinjury was 7.9% among patients treated with PRP, compared to 26.8% of those treated without PRP [49].

It is important to emphasize that, with the exception of two cases of infection, no complications have been reported from the use of PRP. Bergeson et al. [17] showed an infection rate of 12% among patients treated with fibrin matrix rich in platelets without leucocytes compared to 0% in the control group. However, this difference did not reach statistical significance, and no difference in the rates of infection or complication rates was found in the remaining studies.

Although clinical studies have produced conflicting results, data on PRP suggest a beneficial effect on healing process when applied during rotator cuff repair. The stratified analysis of small or medium lesions showed a significantly lower retear rate in the PRP group. Therefore, it currently seems that PRP may improve healing of arthroscopically repaired small and medium rotator cuff lesions, which appear more prone to a biological response to treatment with growth factors.

Further prospective randomized controlled trials (level 1 evidence) are necessary to define the role of PRP in healing of rotator cuff repair.

### 3.3. PRP Injections for Rotator Cuff Tendinopathy

Injections of PRP have gained popularity in the treatment of tendinopathy because of their promoting effects on tendon cell proliferation, collagen synthesis, and vascularization, which have been shown in animal and* in vitro* studies [34, 50].

In spite of this popularity and increasing use in clinical settings we have found only two controlled randomized trials evaluating the use of PRP injections in rotator cuff tendinopathy [19, 20].

These studies have reported controversial results on the effectiveness of the use of PRP injection in chronic rotator cuff tendon diseases.

The systems of PRP preparation were not the same among trials and different treatment protocols were used (single or double PRP injections). Furthermore, the presence of some bias including the concomitant standard exercise program and the needle stimulus effect can have influenced the results of studies. The nature of rotator cuff disease was also not the same throughout the studies and patients refractory to physical therapy and corticosteroid injection seem to have a benefit from the PRP use.

Extrinsic and intrinsic factors including anatomical problems, joint kinematics alterations, and age- and vascularity-related degenerative changes may play a role in developing such disease.

More studies with a high level of evidence are required to validate the role of PRP injections in the subacromial space for treatment of rotator cuff diseases.

## 4. Stem Cells

### 4.1. Definition

Stem cells are defined as unspecialized cells with a self-renewal potential, which are able to differentiate into various adult cell types. The most common stem cell sources are embryonic and adult stem cells.

Embryonic stem cells are truly pluripotent; that is, they are able to differentiate into all derivatives of the three primary germ layers: ectoderm, endoderm, and mesoderm.

In contrast to embryonic stem cells, multipotent adult stem cells are characterized by a differentiation potential restricted to tissues of 1 germ layer. Those which can differentiate into various forms of mesenchymal tissue (i.e., bone, tendon, cartilage, and muscle) are termed mesenchymal stem cells (MSCs).

Most of the clinical related stem cells research to date has focused on adult stem cells rather than embryonic stem cells, as the latter are associated with numerous regulatory and ethical constraints.

### 4.2. Mesenchymal Stem Cells

MSCs are progenitor cells that have the capacity to self-renew and differentiate into several different mesenchymal tissues including muscle, fat, bone, ligament, tendon, and cartilage [51]. For this reason, MSCs can theoretically be stimulated to undergo differentiation to a preferred lineage (osteocytes, chondrocytes, tenocytes, and adipocytes), thus recreating a specific tissue for therapeutic use [51].

### 4.3. Source of MSCs

Bone marrow is the main source of MSCs for rotator cuff healing and can be easily accessed by surgeons to harvest cells, the extraction and culture techniques of which, as well as the conditions for propagation, have been extensively defined. For these reasons, bone marrow-derived MSCs (BMSCs) may have valid clinical use, and there is evidence showing that BMSCs can be manipulated to differentiate into a tenogenic lineage and produce tendon tissue when exposed to the appropriate stimuli.

The iliac crest is the most common site for MSC harvesting, although a number of other sources have been recently identified. Recent research performed by Mazzocca et al. [52] demonstrated that MSCs can be successfully and safely harvested from the proximal humerus during arthroscopic rotator cuff repair in humans and thus potentially applied to the repair site of the same patient to augment tendon-to-bone healing. In another study, authors characterized the harvested cells as BMSCs and induced differentiation in tenocyte-like cells by treatment with insulin [53]. This group has shown that it is possible to extract culture and differentiate stem cells into tendon cells in humans.

Beitzel et al. [54, 55] showed that arthroscopic bone marrow aspiration from the proximal humerus is a reproducible technique and yields reliable concentrations of MSCs. These studies demonstrate that BMSCs can be harvested avoiding an additional surgical site for aspiration (i.e., iliac crest) or a second operative procedure, making future use of MSCs in arthroscopic rotator cuff surgery easy.

MSCs can also be collected from other sources, such as adipose tissue; this can be easily accessible although its cells have an apparently reduced ability to differentiate compared to BMSCs [56].

Tendon derived stem cells (TDSCs) are considered of extreme interest for rotator cuff repair enhancement. Existence of TDSCs has been first shown in murine patellar and human hamstring tendons by Bi et al. [57]. More recent* ex vivo* studies confirmed TDSCs isolation from animal and human rotator cuff tissues. Tsai et al. [58] showed on 5 patients that cells harvested from the rotator cuff tendon could be successfully isolated and differentiated into cells with MSCs characteristics. In 2013, Randelli et al. [59] confirmed the existence of new stem cell populations in shoulder tissues; samples from human supraspinatus tendon and human long head of the biceps tendon were collected during arthroscopic rotator cuff repairs from 26 patients. Morphology, self-renewal capacity, immunophenotype, gene and protein expression profiles, and differentiation capacity were evaluated and resulted in characterization of two new types of human stem cells. In the same year, Utsunomiya et al. [60] isolated and characterized MSCs from four shoulder tissues: synovium of glenohumeral joint, subacromial bursa, rotator cuff tendon, and enthesis at greater tuberosity, obtained from shoulder joint of 19 patients undergoing arthroscopic rotator cuff repair, suggesting that subacromial bursa is a good candidate for the source of MSCs in rotator cuff tears.

Recently, Song et al. [61] isolated MSCs from bursa tissue associated with rotator cuff tendons from five patients undergoing rotator cuff surgery and characterized them for multilineage differentiation* in vitro *and* in vivo*. These results showed in animal models that the cells isolated from bursa tissue exhibited MSCs characteristics and high proliferative capacity, and differentiated toward cells of mesenchymal lineages (osteoblasts, tenocytes, and fibrochondrocytes) with high efficiency suggesting that bursa, a tissue usually discarded during rotator cuff tear repairs, is a new abundant source of MSCs with a high potential for application.

### 4.4. Animal Studies

The use of MSCs to enhance tendon regeneration has been examined in multiple animal models of tendon healing. MSC can be applied directly to the site of injury or can be delivered on a suitable carrier matrix, which functions as a scaffold while tissue repair takes place. In an attempt to augment tendon-bone healing in a rat rotator cuff repair model, Gulotta et al. [62] in 2009 conducted a case-control study on 80 rats that underwent unilateral detachment and repair of the supraspinatus tendon. BMSCs obtained from long bones of 10 rats were applied to the repair site. No significant differences were observed with the control group, in which the rotator cuff repair was not followed by stem cells injection. Authors concluded that MSCs alone are not sufficient to improve tendon-to-bone healing in a rotator cuff model as the repair site may lack the cellular and/or molecular signals needed to induce appropriate differentiation of the transplanted cells, suggesting that additional differentiation factors may need to be combined with this cell-based therapy to be effective. Knowledge of the biological signaling events that lead to the formation of the natural enthesis suggests candidate molecules that could be used in combination with MSCs to augment the repair site. Therefore, in later studies, Gulotta et al. [63, 64] examined various types of transduced MSCs with the aim of driving the healing process toward regeneration rather than repair of the tendon-bone structure. Three controlled laboratory studies showed that transducing cells with scleraxis or membrane type 1 matrix metalloproteinase improved histological quality and biomechanical strength as early as 4 weeks after repair, whereas transducing the cells with BMP-13 did not achieve favorable results.

Yokoya et al. [65] studied the implantation of a polyglycolic acid sheet seeded with cultured autologous BMSCs in a complete infraspinatus lesion created in a rabbit model. Sixteen weeks after the implantation, an increased production of type I collagen and an increment of the mechanical strength was seen as compared with both a nonaugmented control and a nonloaded scaffold group.

Kim et al. [66] harvested BMSCs from the iliac crest of 2 rabbits and cultured and seeded them on a tridimensional open-cell polylactic acid scaffold. A similar scaffold without stem cells was implanted on the contralateral shoulder as control. This study showed that BMSCs survived for 2, 4, and 6 weeks within the scaffold and type I collagen expression was increased in the scaffold with BMSCs as compared with control.

Shen et al. [67] used a knitted silk-collagen scaffold, loaded with allogenous Achilles tendon stem cells, to augment a rotator cuff repair in rabbits, and compared these with repairs augmented with a nonloaded scaffold. No reject reactions were observed and increased fibroblastic cell ingrowth and reduced infiltration of lymphocytes within the implantation site were observed in the treatment group after 4 and 8 weeks. Morphological evaluation performed after 12 weeks showed an improvement in structural and mechanical proprieties, as compared with control.

### 4.5. Clinical Studies

Up to now, only one cohort study has evaluated the safety of clinical application of MSCs in shoulder surgery. In this study Gomes et al. [68] investigated the effects of bone marrow mononuclear cells (BMMCs) in 14 patients with complete rotator cuff tears, suggesting that BMMCs are a safe and promising alternative to other biological approaches to enhance tissue quality in affected tendons.

Autologous BMMCs were harvested from the iliac crest prior to the surgical repair and subsequently injected into tendon borders after being fixed down by transosseous stitches. The BMMC fractions were obtained by cell sorting and resuspended in saline enriched with 10% autologous serum. Each patient was monitored for a minimum of 12 months, and University of California, Los Angeles (UCLA), scores improved on average from 12 to 31, and magnetic resonance imaging showed tendon integrity in all 14 patients. No control group was included in this study, but for this procedure, overall rates of rerupture during the first postoperative year range from 25% to 65%, depending on lesion diameter. Only 1 patient in the following year relapsed with loss of strength and pain. Unfortunately, only 14 patients were enrolled in this study, making it difficult to determine the efficacy of BMMCs as an adjunct to cuff repair at this time. However, these results suggest that BMMC therapy is a safe treatment that has potential to enhance tendon repair. Further research will be critical to better investigate the use of this biologic approach.

## 5. Conclusions

Several regenerative approaches have been investigated to augment tendon healing after arthroscopic cuff repair.

The ability of numerous growth factors to affect tendon healing has been extensively analyzed* in vitro *and* in animal models, *showing promising results. However there is still no study on the use of growth factors in the treatment of rotator cuff on human.

Different delivery systems for these factors, including simple injection, coated sutures, fibrin sealants, heparin-fibrin delivery systems, collagen, and hyaluronic acid sponges, are being tested. PRP is a whole blood fraction which contains several growth factors. Different PRP formulations exist: leucocyte-poor and leucocyte-rich, activated and unactivated. Moreover, PRP can be administrated either with a simple injection or in a fibrin-matrix clot. Clinical trials using different autologous PRP formulations after rotator cuff tear repairs have provided controversial results.

However, favourable structural healing rates have been observed for surgical repair of small and medium rotator cuff tears.

Cell-based approaches have also been suggested to enhance tendon healing. Bone marrow is a well known source of MSCs; recently,* ex vivo* human studies have isolated and cultured distinct populations of MSCs from rotator cuff tendons, long head of the biceps tendon, subacromial bursa, and glenohumeral synovia. A single clinical study has been conducted on stem cell-based therapies for rotator cuff healing, proving the injection of bone marrow mononuclear cells to be safe. Clinical research regarding the use of MSCs in shoulder surgery is very limited. Further basic and clinical investigations are required until a procedure can be defined for the routine use of these cells in shoulder surgery.

## Figures and Tables

**Table 1:** Controlled clinical studies investigating the use of PRP in rotator cuff lesions.

Surgical use of PRP in arthroscopic rotator cuff repair					
Author	Evidence	PRP formulation	Surgical technique	Number of patients	Comments
Randelli et al. (2011) [5]	Level 1 Randomized controlled	Injectable PRP (GPS system)	Single row	53	Better clinical outcomes at 3 mo; better clinical outcomes at 12, 24 months for smaller tears with PRP
Ruiz-Moneo et al. (2013) [6]	Level 1 Randomized controlled	Injectable PRP (PRGF Endoret system)	Double row	63	No differences in rotator cuff healing or function at 1 year
Antuña et al. (2013) [7]	Level 2 Randomized controlled	Injectable PRP (Vivostat system)	Single row	28	No differences in clinical outcomes and healing rate at 2 years
Charousset et al. (2014) [8]	Level 3 Case control	Injectable PRP (GPS system)	Double row	70	No differences in cuff healing or function at 2 years A significant advantage for the L-PRP patients in terms of smaller iterative tears
Gumina et al. (2012) [9]	Level 1 Randomized controlled	Suturable PRP (RegenKit-THT system)	Single row	76	Lower retear in the PRP group; no differences for clinical outcomes
Jo et al. (2011) [10]	Level 2 Prospective cohort	Suturable PRP (COBE spectra system)	Transosseous equivalent	42	Trend for lower re-tearing in the PRP group; no differences for recovery and function
Jo et al. (2013) [11]	Level 1 Randomized controlled	Suturable PRP (COBE spectra system)	Transosseous equivalent	48	Lower retear and function at 1 year in the PRP group
Zumstein et al. (2014) [12]	Level 1 Randomized controlled	Suturable PRP (PRF process)	Transosseous equivalent	20	Increased vascularization for cuff tears with PRP
Castricini et al. (2011) [13]	Level 1 Randomized controlled	Suturable PRP (Cascade system)	Double row	88	No difference for clinical outcomes at 16 months; better restoration of footprint in PRP group Lower retear using the chi-square test for binomial in Arnoczky [14] analysis
Rodeo et al. (2012) [15]	Level 2 Randomized controlled	Suturable PRP (Cascade system)	Single OR double row/transosseous equivalent	67	No difference in tendon healing, tendon vascularity, and clinical scores at 1 year
Barber et al. (2011) [16]	Level 3 Case-control study	Suturable PRP (Cascade system)	Single row	40	Lower retear in the PRP group; better healing for smaller tears with PRP
Bergeson et al. (2012) [17]	Level 3 Cohort study	Suturable PRP (Cascade system)	Single or double row	37	Higher retear rate in patients with at-risk rotator cuff tears with PRFM; no difference in functional outcome scores Historical control group
Weber et al. (2013) [18]	Level 1 Randomized controlled	Suturable PRP (Cascade system)	Single row	60	No difference in perioperative morbidity, clinical outcomes, or structural integrity

PRP injections for rotator cuff tendinopathy					
Author	Evidence	PRP intervention	Control intervention	Number of patients	Comments

Rha et al. (2013) [19]	Level 1 Randomized controlled	2 PRP (3 mL) injections at a 4-week interval	2 dry needling procedures at a 4-week interval	39	PRP was superior with respect to pain, function, and range of motion over a 6-month period
Kesikburun et al. (2013) [20]	Level 1 Randomized controlled	1 injection of PRP (5 mL)	1 injection of saline solution (5 mL)	40	No difference for quality of life, pain, disability, and range of motion at 1 year
